# Inflammatory markers and blood glucose are higher after morning vs afternoon exercise in type 2 diabetes

**DOI:** 10.1007/s00125-025-06477-5

**Published:** 2025-06-28

**Authors:** Martin J. Keller, Aidan J. Brady, Jonathan A. B. Smith, Mladen Savikj, Kirstin MacGregor, Maxence Jollet, Sofia B. Öberg, Carolina Nylén, Marie Björnholm, Anette Rickenlund, Marcus Carlsson, Kenneth Caidahl, Anna Krook, Nicolas J. Pillon, Juleen R. Zierath, Harriet Wallberg-Henriksson

**Affiliations:** 1 Department of Physiology and Pharmacology, Karolinska Institutet, Stockholm, Sweden; 2 Department of Molecular Medicine and Surgery, Karolinska Institutet, Stockholm, Sweden; 3 Department of Breast, Endocrine Tumours and Sarcoma, Karolinska University Hospital, Stockholm, Sweden; 4 Department of Clinical Physiology, Karolinska University Hospital, Stockholm, Sweden

**Keywords:** Circadian biology, Continuous glucose monitoring, Diet, High-intensity interval exercise, Inflammation, Sex differences, Type 2 diabetes

## Abstract

**Aims/hypothesis:**

The aim of this study was to investigate effects of time of day on glucose management in individuals with type 2 diabetes undertaking high-intensity interval exercise. Additionally, the association between regular eating behaviour and mean amplitude of glycaemic excursions was examined. Specifically, the primary outcome was to determine the effect of the intervention on 24 h glucose levels.

**Methods:**

A crossover trial was conducted, comprising 12 men and 12 women with type 2 diabetes and 12 men and 12 women without diabetes. Participants performed high-intensity interval exercise sessions in the morning (09:00 hours) or afternoon (16:00 hours) on separate days at least 7 days apart. Standardised meals were provided the day before exercise, on the day of exercise and on the day after exercise. Continuous glucose monitoring was used to estimate blood glucose levels.

**Results:**

The 24 h glucose profile did not differ between morning and afternoon exercise across cohorts. However, morning exercise increased blood glucose during the 2 h post-exercise period in men (*p*<0.05) and women (*p*<0.01) with type 2 diabetes, but blood glucose was unaltered following afternoon exercise. Glycaemic variability (assessed using the mean amplitude of glycaemic excursions) was reduced during the 3 day meal intervention in men (*p*<0.001) and women (*p*<0.05) with type 2 diabetes, but not in individuals without diabetes. Participants exhibited higher morning cortisol levels (*p*<0.001) compared with afternoon cortisol levels, independently of diagnosis. Individuals with type 2 diabetes exhibited higher levels of the inflammation marker C-reactive protein (*p*<0.001) and the heart failure marker NT-proBNP (*p*<0.001) in the morning than in the afternoon.

**Conclusions/interpretation:**

In type 2 diabetes, afternoon high-intensity interval exercise appears to be more effective than morning high-intensity interval exercise for maintaining glucose management. Further research is needed to explore how elevated morning cortisol levels and inflammatory markers influence the exercise response and affect glucose regulation. Additionally, consistent meal timing and controlled energy intake are recommended for reducing the mean amplitude of glycaemic excursions.

**Trial registration:**

ClinicalTrials.gov NCT05115682.

**Graphical Abstract:**

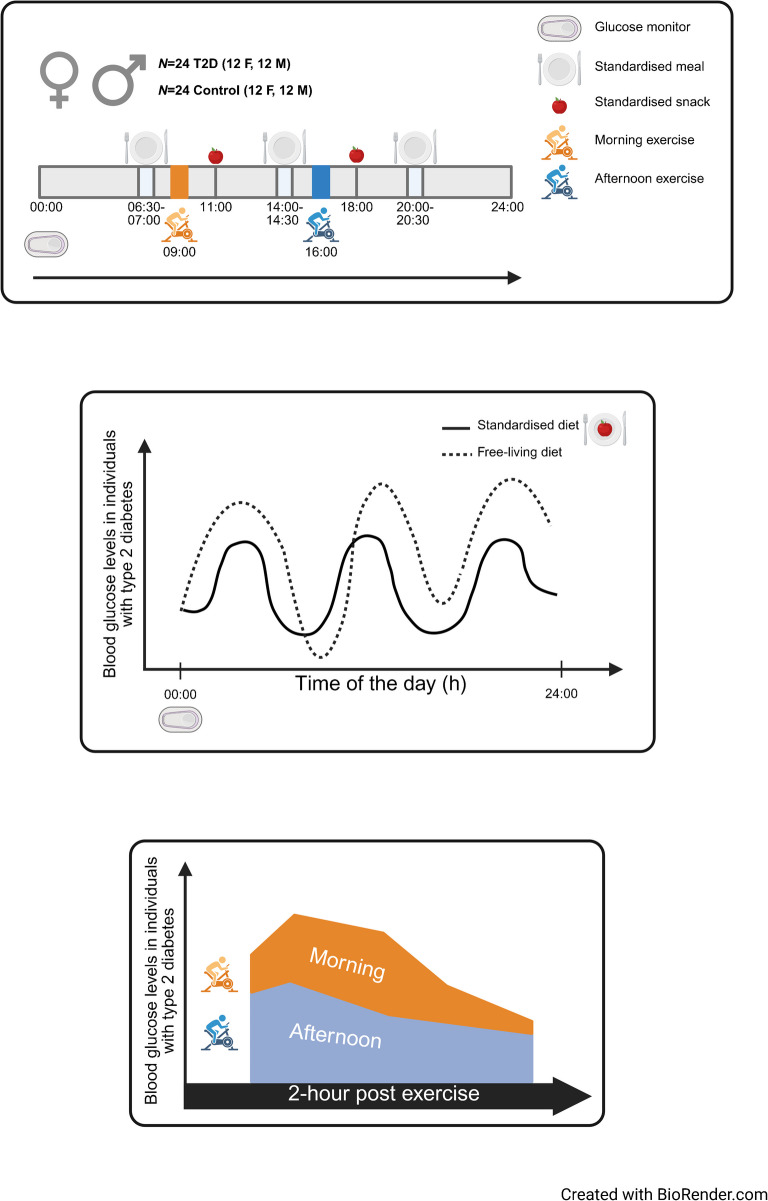

**Supplementary Information:**

The online version of this article (10.1007/s00125-025-06477-5) contains peer-reviewed but unedited supplementary material.


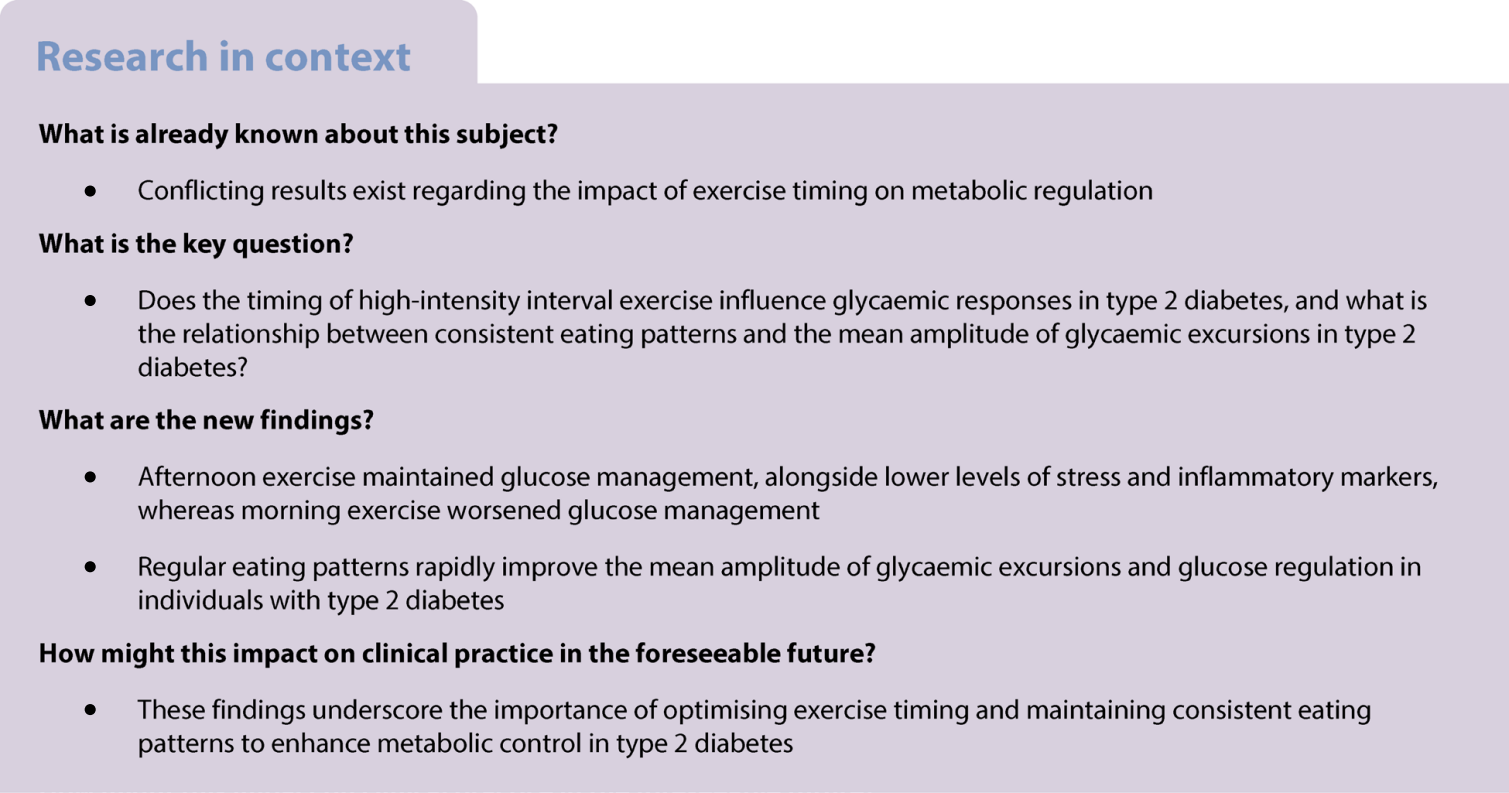


## Introduction

Lifestyle interventions, particularly those relating to exercise and diet, are fundamental to the prevention and management of metabolic diseases, including type 2 diabetes [1, 2]. However, the optimal strategies for implementing these interventions, especially regarding the timing of exercise and eating patterns, remain poorly understood. Integrating an understanding of circadian biology into exercise and dietary recommendations may pave the way for more effective metabolic disease management.

The circadian system plays a pivotal role in glucose homeostasis, with disruptions to circadian rhythms impairing glucose sensitivity [3]. Recent research has revealed a time-of-day effect for exercise-mediated glycaemic control. For instance, men with type 2 diabetes exhibited improved glucose management when performing high-intensity interval training in the afternoon compared with the morning over a 2 week period [4]. Similarly, in a 12 week intervention, men at risk of or diagnosed with type 2 diabetes showed increased peripheral insulin sensitivity following afternoon exercise, but morning exercise failed to elicit similar improvements [5]. Additionally, evening exercise, but not morning exercise, improved fasting glucose levels and night-time metabolic control in men with overweight or obesity [6]. Timing of exercise in alignment with circadian biology may represent an effective approach to optimising glycaemic control, highlighting the need for further research to elucidate the mechanisms underlying these time-of-day effects. However, exercise of low to moderate intensity in the morning or afternoon in individuals with type 2 diabetes [7] or obesity [8] showed no significant differences, highlighting the additional role of exercise intensity. Collectively, while some evidence suggests that afternoon exercise may confer greater benefits for glycaemic control in individuals with type 2 diabetes or obesity, inconsistent findings indicate that further research is needed to confirm these effects.

Studies investigating the time-of-day effects of exercise in individuals with type 2 diabetes or obesity have predominantly involved only men [4–6]. Additionally, men with type 2 diabetes have an exacerbated inflammatory response to an acute high-intensity exercise bout [9], but whether this occurs in women remains incompletely characterised. This represents a critical gap in the literature, as men and women exhibit distinct physiological responses to exercise due to hormonal and metabolic differences, including the influence of oestrogen on glucose metabolism [10–12]. Without examining these differences, findings from male-dominated studies cannot be generalised, limiting the development of personalised and effective treatment strategies for managing type 2 diabetes in women.

The 2025 Standards of Care for Diabetes published by the ADA underscore eating management as an essential component of diabetes care [13]. Despite this emphasis, modern society is characterised by increasingly erratic eating patterns [14], which are negatively correlated with the mean amplitude of glycaemic excursions (MAGE) in normoglycaemic individuals [15]. The impact of eating behaviours on MAGE in individuals with type 2 diabetes has been investigated predominantly within the context of weight-loss interventions [16]. Given the established direct correlation between MAGE and cardiovascular events [17], maintaining a low MAGE is particularly critical for individuals with type 2 diabetes. Furthermore, consistent meal timing improved glycaemic control in people at risk for type 2 diabetes [18]. These observations highlight the need to explore how structured eating patterns beyond weight-loss interventions can be leveraged to optimise glycaemic variability and reduce cardiovascular risk in individuals with type 2 diabetes.

Our previous research demonstrated that afternoon high-intensity interval exercise improved glucose management in men with type 2 diabetes, while morning sessions worsened blood glucose management [4]. However, this previous study did not account for food intake or evaluate the acute glucose response to a single bout of high-intensity interval exercise. Given that food intake can affect glucose management throughout the day, meal timing should also be considered. Here, we hypothesised that different timings of a single session of high-intensity interval exercise would elicit distinct glycaemic responses in men and women with type 2 diabetes when food intake is controlled. Additionally, we hypothesised that adherence to regular eating patterns would be positively associated with lower glycaemic variability in individuals with type 2 diabetes.

## Methods

### Study design and participants

This clinical trial was conducted at the Karolinska University Hospital between 2021 and 2024. Ethical approval was obtained from the Swedish Ethical Review Authority (2019–04747), and the trial was prospectively registered in the Clinical Trial Registry (ClinicalTrials.gov NCT05115682). The study adhered to the ethical principles outlined in the Declaration of Helsinki, and all participants provided written informed consent prior to participation.

A crossover design was employed, in which participants completed morning and afternoon high-intensity interval exercise sessions on separate days at least 7 days apart. The study comprised two visits consisting of a pre-intervention day (baseline), day 1 (meal intervention), day 2 (meal and exercise intervention), day 3 (meal intervention) and a post-intervention day (baseline). Interstitial glucose levels were continuously monitored using continuous glucose monitors (CGMs) from 72 h before to 72 h after each exercise session. To standardise dietary intake, participants were provided with standardised meals and instructed to adhere to a fixed eating schedule comprising three main meals and one snack for women or two snacks for men per day, with any deviations recorded in a food diary.

### Clinical cohorts

Participants were recruited prospectively through advertisements in media such as newspapers, diabetes newsletters and various social media platforms in the larger Stockholm area. Regional and socioeconomic factors were not accounted for in the recruitment process. Initial eligibility screening was conducted via phone, email or using an online screening questionnaire via the REDCap application [19], and included assessments of medical history, age and BMI. To minimise potential genetic and physiological variability, we intentionally recruited a sample of individuals who described themselves as being of European descent; while this approach aims to reduce heterogeneity, it also limits the generalisability of our findings and thus the results may not represent the broader population’s metabolic responses to exercise timing. The study design was unique, and no directly comparable cohorts were available to support a formal a priori power calculation. Thus, an estimation based on anticipated biological effects was performed. Assuming the primary source of variability stemmed from CGM measurements, with an estimated SD of approximately 1.5 mmol/l, we calculated that approximately 15 participants would be required to detect a 1 mmol/l difference in glucose levels with 80% power and a two-sided alpha value of 0.05. As we anticipated greater variability in the glucose responses among individuals with type 2 diabetes, the final sample size was 24 participants per group.

Participants were included if they met the following criteria: men and women (sex was self-reported) aged 45–68 years, with a BMI between 23 and 33 kg/m^2^. All the women were postmenopausal. For individuals with type 2 diabetes, eligibility required that diagnosis according to the diagnostic criteria established by the Swedish National Board of Health and Welfare [20] was confirmed at least 12 months previously. Control participants were required to have HbA_1c_ levels below 5.7%.

**Fig. 1:**
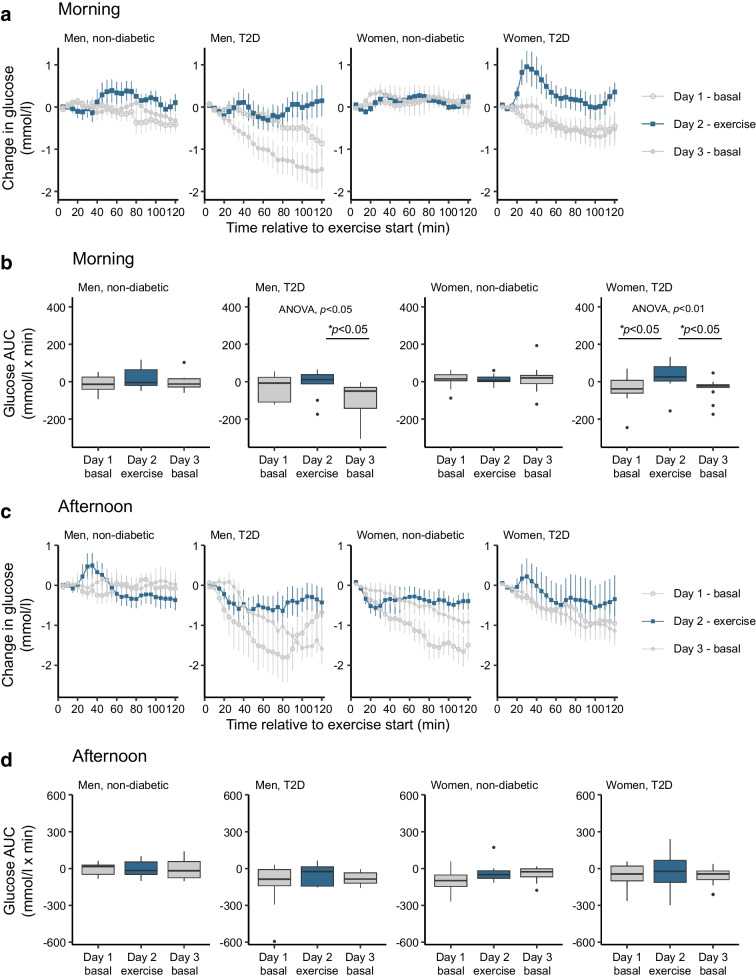
Elevated blood glucose after morning exercise in individuals with type 2 diabetes (T2D). (**a**, **c**) Glucose levels (means ± SEM) were normalised to the exercise start as described in the Methods, and are presented for the 120 min period following the start of exercise for day 1 (pre-exercise day, grey open circles), day 2 (exercise, blue squares) and day 3 (post-exercise day, grey closed circles). (**b**, **d**) The AUC was calculated as described in the Methods and is displayed as box-and-whisker plots. Box and whisker plots present the median, first quartile (Q1), and third quartile (Q3). The whiskers extend to the largest and smallest values within 1.5 times the interquartile range (IQR) from the quartiles. Outliers are defined as data points that lie beyond 1.5 times the IQR above Q3 or below Q1. The results of statistical analyses comprise *p* values derived from the ANOVA (as shown) and post hoc comparisons of exercise responses performed using a linear mixed-effects model as outlined in the Methods: *p<0.05

The exclusion criteria were prior or current insulin therapy, not being able to use a cycle ergometer, active smoking or smoking cessation within the past 6 months, cardiovascular conditions, blood-borne diseases, rheumatic illness, malignancies, other systemic disease, use of anti-anxiety medications, or inability to comply with the meal plan. Following screening, a total of 48 participants were enrolled; these participants were evenly distributed among four groups: 12 women with type 2 diabetes, 12 women without type 2 diabetes, 12 men with type 2 diabetes and 12 men without type 2 diabetes (see electronic supplementary material [ESM] Fig. 1).

Nine women and 11 men with type 2 diabetes were being treated with metformin, while six women were prescribed glucagon like peptide-1 agonists. Additionally, three women and two men with type 2 diabetes were being treated with sodium-glucose co-transporter-2 inhibitors. Seven women and six men with type 2 diabetes were receiving cholesterol-lowering statin medication, while only one man in the control group was receiving statin treatment. For BP management, seven women and five men with type 2 diabetes were being treated with BP medication, compared with two women and two men in the control group.

### Cardiopulmonary exercise testing

Each participant completed an incremental exercise test to volitional exhaustion on a stationary cycle ergometer (eBike III comfort ergometer, GE Healthcare). The protocol began with a 3 min warm-up of unloaded cycling, after which resistance was increased at a rate of 10–20 W/min, tailored to each participant’s habitual exercise level and capability. Peak workload was recorded as the load achieved at the point of test termination.

During the cardiopulmonary exercise testing, expired air was analysed continuously using an automated breath-by-breath system (Vyntus CPX metabolic cart; Vyaire Medical, Mettawa, IL, USA). Peak oxygen uptake ($\dot{V}{\text{O}}_{2\text{peak}}$) was taken as the highest 20 s oxygen uptake recorded during the test. The anaerobic threshold was identified using the ventilatory equivalent method, defined as the point at which the ventilatory equivalent for oxygen ($\dot{V}\text{E}$/$\dot{V}{\text{O}}_{2}$) increased without a corresponding rise in the ventilatory equivalent for carbon dioxide ($\dot{V}\text{E}$/$\dot{V}{\text{CO}}_{2}$). The deflection point in the $\dot{V}{\text{CO}}_{2}$ vs $\dot{V}{\text{O}}_{2}$ relationship was used to confirm the threshold [21]. The heart rate was monitored continuously using a 12-lead ECG (EC Sense ECG; Cardiolex Medical, Stockholm, Sweden). Ratings of perceived exertion (using the Borg 6–20 scale [22]) and the perception of dyspnoea were recorded.

### Ultrasound

During the screening phase, a Vivid E95 ultrasound system (GE Healthcare) was used for transthoracic echocardiographic examination of the heart (using a 4Vc-D transducer, frequency 4.0–10.0 MHz) and for scanning of arterial neck vessels (using a 9L-D transducer, frequency 3.0–8.0 MHz, or an 11L-D 9L-D transducer, frequency 3.0–11.0 MHz, as applicable for best image quality). Investigations followed the standards established by the American Society of Echocardiography and the European Association of Cardiovascular Imaging [23]. Tissue images and Doppler flow signals were recorded, ensuring optimal measurement accuracy by aligning the ultrasound beam with blood flow or myocardial motion. Images were stored on a Viewpoint server (Viewpoint 6, version 6.12; GE Healthcare). Trained physicians analysed the images and assessed participant suitability. Individuals with advanced atherosclerotic plaque in the carotid vessels were excluded from further participation.

### Clinical chemistry

One week prior to the intervention, participants provided an overnight fasted blood sample at the Karolinska University Hospital for routine clinical chemistry analysis. The panel includes standardised assays performed at Karolinska University Laboratory. The following variables were measured: HbA_1c_, N-terminal prohormone of brain natriuretic peptide (NT-proBNP), troponin T, insulin, C-peptide, glucose, C-reactive protein (CRP), total cholesterol, LDL-cholesterol, HDL-cholesterol, triglycerides, alanine aminotransferase, aspartate aminotransferase, apolipoprotein A1, apolipoprotein B, creatinine, potassium and haemoglobin. During each experimental trial, venous blood samples were collected before, immediately after and 1 h after the exercise session for analysis of cortisol, CRP, NT-proBNP, insulin and glucagon.

### Continuous glucose measurement

Participants were fitted with a Dexcom G6 CGM (Dexcom, San Diego, CA, USA), and glucose data were recorded every 5 min from approximately 72 h before exercise until approximately 72 h post-exercise. The CGM data from all participants were pre-processed individually, annotated and then merged to ensure standardisation of all time points. Data were grouped into morning and afternoon exercise sessions, with days defined as 24 h cycles starting at 06:00 hours on the day prior to the intervention. Glucose readings were categorised into 5 min intervals, and means for each interval were calculated.

To compare effects of morning and afternoon exercise, analyses of CGM data for each participant were performed relative to the time of exercise initiation. Glucose values were normalised to the mean glucose level measured within a 10 min window around the exercise start time (−5 to +5 min) for each individual and condition. This normalisation accounted for baseline glycaemic differences across groups and facilitated direct comparisons of glycaemic responses to exercise.

Glycaemic variability metrics, including AUC and MAGE were calculated using the R statistical software (www.r-project.org) package iglu version 4.1.6 [24].

### Standardised meals

Standardised meals were provided during the meeting for CGM fitting. Participants were given a choice of six meal options and four snack options, all designed to meet standardised macronutrient profiles. The meals contained approximately 2259 kJ (540 kcal), comprising 50 g carbohydrates, 25 g protein and 25 g fat. Snacks provided 950 kJ (227 kcal), consisting of 24 g carbohydrates, 16 g protein and 7 g fat. Participants were instructed to adhere to a fixed meal schedule, consuming breakfast between 06:30–07:00 hours, lunch between 13:30–14:00 hours and dinner between 19:30–20:00 hours on the day before exercise, the day of exercise and the day after exercise. Snacks were consumed at 11:00 hours and 18:00 hours. Participants were required to consume identical meals for breakfast and lunch and a different meal for dinner on all meal intervention and meal and exercise intervention days, thus ensuring that the macronutrient composition was consistent between trials.

### Exercise session

Participants completed a crossover trial consisting of a single high-intensity interval exercise session conducted in a clinical setting under the supervision of trained exercise physiologists and licensed physicians. The exercise session was performed either in the morning (09:00 hours) or afternoon (16:00 hours), with a minimum wash-out period of 7 days between sessions. Exercise sessions were performed on the same stationary cycle ergometer (eBike III comfort ergometer) as used during the cardiopulmonary exercise testing, with the saddle height adjusted to match the prior setting. The high-intensity interval exercise protocol began with a 7 min warm-up, starting at 50 W for men and 30 W for women, with a cadence of 75 rev/min. Resistance was increased incrementally each minute until 70% of the peak workload recorded during the cardiopulmonary exercise testing was reached, followed by a 1 min recovery period at the starting resistance (50 or 30 W). Participants then performed six intervals of 1 min at 100% of peak workload, followed by 1 min of recovery at 50 W (men) or 30 W (women), at a cadence of 75 rev/min. The exercise protocol concluded with a 3 min cool-down period at the starting resistance (50 or 30 W) and the same cadence. While the resistance was different between the sexes during the warm-up and recovery periods, the mean rating of perceived exertion was similar between men and women (16±1 and 15±1 arbitrary units, respectively), regardless of metabolic status or time of day. BP was measured before, during and after the exercise session. Heart rate was continuously monitored using a 12-lead ECG (EC Sense ECG). Participants were fitted with a peripheral i.v. cannula inserted into a superficial vein for repeated blood sampling. Blood samples were collected before exercise, immediately post-exercise and at 1 h post-exercise.

### Statistical analysis

Statistical analyses were conducted using R software (version 4.4.1, www.r-project.org). A two-way ANOVA was used to examine the main effects of type 2 diabetes, sex and the interaction of these parameters on the outcomes of interest. The primary outcome was the effect of time of day on glucose management in relation to high-intensity interval exercise following standardised meals. The secondary outcome was to determine the effect of standardised meals on glucose management. Additionally, we determined the effect of exercise at different times of the day on metabolic markers, including stress and inflammatory markers. The normality of data distribution was assessed using the Shapiro–Wilk test, while homogeneity of variance was evaluated using Levene’s test. If assumptions of normality or variance were violated, a Tukey ladder of powers transformation was applied before conducting the ANOVA. Post hoc pairwise comparisons were performed using Tukey’s honestly significant difference (HSD) test. Statistical significance was set at *p*<0.05.

For analysis of glycaemic variability MAGE was calculated during 24 h periods and AUC was calculated 2 h following the start of the exercise. MAGE, AUC and plasma markers of stress and metabolism, were analysed with a linear mixed-effects model. Models were fitted using the lmer function from the R lme4 package (version 1.1-35) to assess the effects of exercise time of day, sex and diabetes status, while accounting for repeated measurements within individuals as a random effect. ANOVA was used to evaluate the significance of these effects. Post hoc pairwise comparisons were performed using estimated marginal means (R emmeans package version 1.10.4), with significance defined as *p*<0.05.

To assess potential carryover effects, a sequence factor (morning → afternoon vs afternoon → morning) as a fixed effect in the linear mixed-effects model was used to evaluate differences in glucose AUC following exercise at different times of day. This analysis was limited to women with type 2 diabetes, as this group showed the strongest intervention effect. The sequence term was not statistically significant (*p*=0.553), indicating no detectable carryover effects. The *p* values for the main effects remained unchanged after including the sequence term, continuing to demonstrate a significant increase in glucose levels following morning exercise, but not afternoon exercise.

## Results

### Participant recruitment

A total of 850 individuals expressed interest in the study in response to various advertising channels. Of these, 775 individuals (91%) were deemed unsuitable based on the inclusion and exclusion criteria. The remaining 75 individuals (9%) were invited to participate in a formal screening process. During the screening, 20 participants (27%) were excluded for reasons such as progressive plaque build-up, ECG abnormalities or HbA_1c_ outside the specified range. An additional seven participants (13%) were excluded during the intervention due to non-adherence to the protocol or adverse reactions to the intervention. Ultimately, 48 participants completed the entire study protocol, representing 6% of the original pool of interested individuals (ESM Fig. 1).

### Participant characteristics

Descriptive characteristics of the study participants are summarised in Table 1. Participants with type 2 diabetes were matched to their respective control group by age, height, weight and BMI. Significant effects of disease status were observed for several metabolic markers: blood glucose, HbA_1c_, C-peptide, total cholesterol, HDL- and LDL-cholesterol, and triglycerides (Table 1). Additionally, sex differences were evident, with women generally exhibiting better glucose management, as indicated by lower blood glucose and HbA_1c_ (Table 1). Men both with and without type 2 diabetes demonstrated clear insulin resistance (HOMA-IR >2.9), whereas women showed values indicative of mild insulin resistance (HOMA-IR >1.4 but <2.9). Plasma insulin levels were higher in men with type 2 diabetes compared with control participants, driven primarily by one participant with an outlier insulin value of 498 pmol/l. No significant differences were observed between groups for resting and peak heart rate or resting and peak systolic BP. However, relative peak workload was reduced in women with type 2 diabetes compared with control participants (*p*<0.05).

**Table 1:** Participant demographic, anthropometric and biochemical characteristics and physiological responses during a cardiopulmonary exercise test to the limit of the participant’s tolerance

	Men		Women		*p* values		
	Control participants	Type 2 diabetes	Control participants	Type 2 diabetes	Sex	Disease	Interaction
*N*	12	12	12	12			
Disease duration (years)		6.18±4.64		6.58±5.48			
Age (years)	56.9±5.2	56.3±5.2	59.6±2.6	59.4±3.2	0.022	0.759	0.864
Height (cm)	183.4±7	181.4±2.2	166.2±6.2	168.1±7.7	<0.001	0.963	0.287
Body mass (kg)	95.5±11.9	92.9±10	78.2±8.9	81.7±11.4	<0.001	0.883	0.333
BMI (kg/m^2^)	28.3±2.5	28.2±3	28.3±2.5	28.9±3.1	0.723	0.760	0.686
WHR	1.00±0.03	0.96±0.06	0.89±0.07	0.93±0.04^*^	<0.001	0.704	0.013
Haemoglobin (g/l)	152±6	154±6	137±8	137±9	<0.001	0.719	0.719
HbA_1c_ (mmol/mol)	36±1	49±7	35±2	45±7^***^	0.029	<0.001	0.772
HbA_1c_ (%)	5.4±0.1	6.6±0.7	5.4±0.1	6.3±0.6^***^	0.029	<0.001	0.772
Glucose (mmol/l)	5.6±0.4	8.1±1.4^***^	5.4±0.5	7±1.2^***^	0.034	<0.001	0.604
Insulin (pmol/l)	86.1±36.1.2	126.2±148.7^***^	47.9±31.3	67.3±48.6	0.004	0.272	0.573
HOMA-IR	3.03±1.19	6.49±7.3	2.01±1.2	2.73±2.3	0.002	0.085	0.531
Troponin T (ng/l)	7±1	9±4	6±1	9±6	0.091	0.058	0.627
NT-proBNP (ng/l)	45±27	32±17	51±34	50±38	0.252	0.314	0.429
CRP (mg/l)	1.6±1.1	1.8±3.1	2.1±2.6	2.1±2	0.638	0.352	0.542
Creatinine (µmol/l)	86±9	82±15	65±8	62±7	<0.001	0.216	0.852
ALAT (µkat/l)	0.59±0.26	0.49±0.12	0.32±0.11	0.51±0.24^**^	0.003	0.082	0.007
ASAT (µkat/l)	0.52±0.08	0.56±0.4	0.35±0.08	0.43±0.12	0.006	0.678	0.049
C-peptide (nmol/l)	0.81±0.21	1.08±0.66	0.61±0.22	0.8±0.34	0.011	0.046	0.567
Total cholesterol (mmol/l)	6.0±1.4	4.8±0.8^*^	5.9±1.2	5.0±1.4	0.827	0.009	0.638
HDL-cholesterol (mmol/l)	1.4±0.3	1.2±0.5	1.9±0.6	1.5±0.3	0.003	0.040	0.504
LDL-cholesterol (mmol/l)	3.8±1.4	2.7±0.5^*^	3.6±1	2.9±1.3	0.969	0.006	0.546
Triglycerides (mmol/l)	1.75±0.8	1.93±1.02	0.79±0.27	1.54±0.97^**^	0.001	0.032	0.066
Apolipoprotein A1 (g/l)	1.54±0.31	1.48±0.32	1.74±0.38	1.56±0.23	0.107	0.212	0.613
Apolipoprotein B (g/l)	1.1±0.43	0.92±0.18	1.03±0.29	0.92±0.33	0.731	0.110	0.731
Resting heart rate (beats/min)	74±11.1	80.4±16.6	75.3±10.2	80.7±6.8	0.816	0.090	0.874
Peak heart rate (beats min)	172.1±11.7	176.6±10.2	168.2±9.8	160.4±11.1	0.002	0.603	0.054
Systolic BP (mmHg)	139.2±16	144.4±15.9	135.4±20.4	134.2±11.2	0.105	0.528	0.637
Peak systolic BP (mmHg)	224.2±18.9	229.2±21.5	205.4±15.6	202.9±21	<0.001	0.825	0.507
Peak $\dot{V}{\text{O}}_{2}$(l/min)	3.00±0.50	2.97±0.38	1.97±0.37	1.83±0.24	<0.001	0.405	0.529
$\dot{V}{\text{O}}_{2\text{peak}}$(ml/kg per min)	31.6±4.3	32.6±6.6	25.9±5.4	22.7±3.9	<0.001	0.468	0.173
Peak workload (W)	267±48.9	258.8±47.3	163.1±31.7	145.2±20.5	<0.001	0.172	0.413
Relative peak workload (W/kg)	2.81±0.45	2.85±0.76	2.14±0.46	1.81±0.35^*^	<0.001	0.105	0.144

Values are means ± SD

ALAT, alanine aminotransferase; ASAT, aspartate aminotransferase

^*^p<0.05 vs control; ^**^p<0.01 vs control; ^***^p<0.001 vs control

### Effect of high-intensity interval exercise on glucose levels

Acute high-intensity interval exercise in the morning on the second day of the dietary intervention altered glucose profiles compared with the first and third intervention days (Fig. 1a). Morning exercise increased post-exercise blood glucose levels (AUC) during the 2 h recovery period for both women (*p*<0.01) and men (*p*<0.05) with type 2 diabetes (Fig. 1b). This effect was not observed in non-diabetic participants. The glucose increase was more pronounced in women with type 2 diabetes compared with men with type 2 diabetes. In contrast, afternoon high-intensity interval exercise did not affect 2 h post-exercise blood glucose levels in either women or men with type 2 diabetes (Fig. 1c, d). High-intensity interval exercise reduced insulin levels (*p*<0.001, ESM Fig. 2) and increased glucagon levels (*p*<0.001, ESM Fig. 2). Women exhibited overall lower levels of insulin (*p*<0.05) and glucagon (*p*<0.001), and a reduced hormonal response to exercise compared with men (*p*<0.05 for insulin; *p*<0.001 for glucagon) (ESM Fig. 2). Additionally, glucagon levels were lower in the afternoon across all groups (*p*<0.05) (ESM Fig. 2). The effects of exercise on insulin and glucagon were consistent across participants with and without type 2 diabetes. The 24 h glucose profile following the exercise bout was not different between trial days (ESM Fig. 3).

### Effect of dietary intervention on continuous glucose measurements

**Fig. 2:**
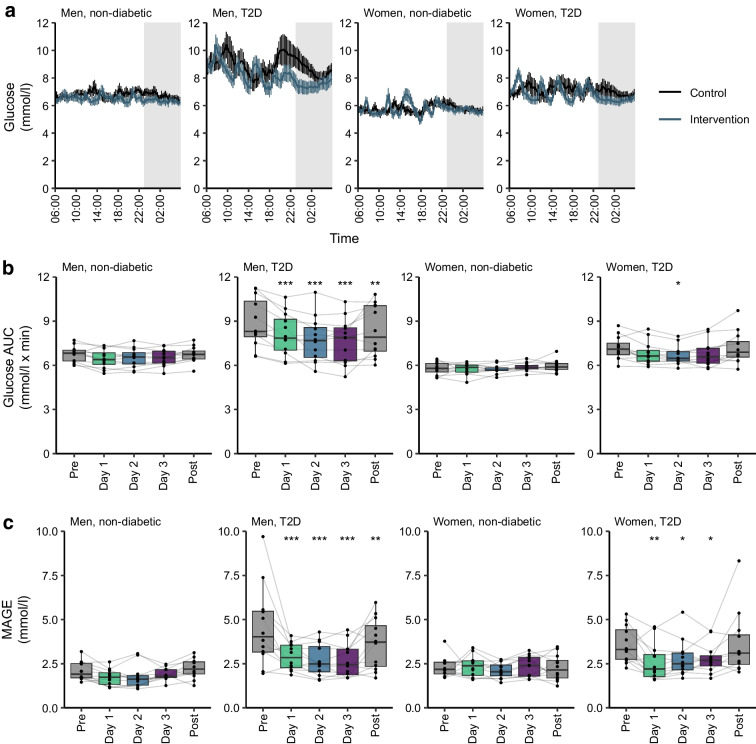
Consistent meal timing improves glycaemic variability in individuals with type 2 diabetes (T2D). (**a**) 24 h glucose concentrations. Control values (black) are the mean of the pre- and post-intervention days. Intervention values (blue) are the mean of the three food control days. White areas are daytime, grey areas are night-time. (**b**) Glucose AUC calculated for 24 h periods for the pre-intervention day, days 1, 2 and 3, and the post-intervention day. (**c**) MAGE calculated for 24 h periods for the pre-intervention day, days 1, 2 and 3, and the post-intervention day. For (**b**) and (**c**), grey bars signify days during which food was not controlled, coloured bars signify days during which food was controlled. The results are shown as box-and-whisker plots, with dots and lines representing the responses of individual participants. Box and whisker plots present the median, first quartile (Q1), and third quartile (Q3). The whiskers extend to the largest and smallest values within 1.5 times the interquartile range (IQR) from the quartiles. Outliers are defined as data points that lie beyond 1.5 times the IQR above Q3 or below Q1. The results of statistical analyses comprise *p* values derived from post hoc comparisons to the pre-intervention day following analysis with a linear mixed-effects model and ANOVA as described in the Methods: *p<0.05, **p<0.01, ***p<0.001

The 3-day dietary intervention period decreased glucose variability in participants with type 2 diabetes, as measured by CGMs, when compared with the pre-intervention day and the post-intervention day (ESM Fig. 3). However, the beneficial effects of dietary intervention were transient, with glycaemic metrics returning to pre-intervention patterns upon resumption of habitual dietary practices. Although the intervention resulted in a reduction in glucose levels (AUC, *p*<0.001) for men with type 2 diabetes on all intervention days, the reduction for women with type 2 diabetes was only apparent on day 2 (*p*<0.05) (Fig. 2a,b). Glycaemic variability (measured by MAGE) was reduced during the intervention for both men (*p*<0.001) and women (day 1: *p*<0.01; days 2 and 3: *p*<0.05) with type 2 diabetes (Fig. 2c). Following the cessation of dietary intervention, AUC and MAGE returned to pre-intervention levels in all groups except men with type 2 diabetes (*p*<0.01).

### Stress and inflammatory response to high-intensity interval exercise

**Fig. 3:**
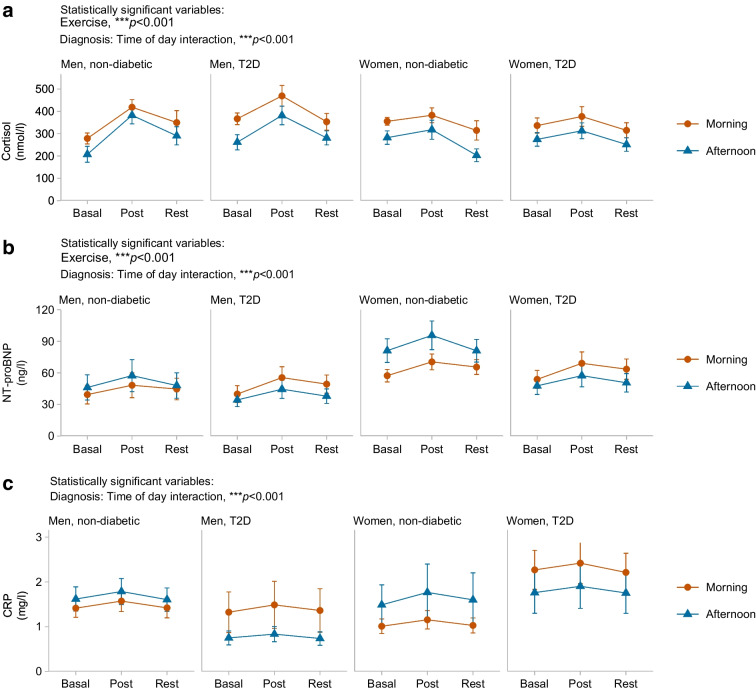
Elevated stress and inflammatory markers in the morning in individuals with type 2 diabetes (T2D). Blood samples were collected immediately before exercise (basal), directly after exercise (post) and at 1 h post-exercise (rest). Values are means ± SEM and are reported for morning (red) vs afternoon (blue) exercise. (**a**) Cortisol levels; (**b**) circulating NT-proBNP levels; (**c**) CRP levels

Cortisol levels were higher in the morning compared with the afternoon across all participant groups (*p*<0.001) and were increased by acute high-intensity interval exercise (*p*<0.001, Fig. 3a). The effect of exercise on cortisol levels did not differ between the morning and afternoon trials. Acute high-intensity interval exercise elevated circulating NT-proBNP levels (*p*<0.001, Fig. 3b). Participants with type 2 diabetes had higher CRP and NT-proBNP levels in the morning, whereas non-diabetic participants showed higher levels in the afternoon (Fig. 3b, c).

## Discussion

This study provides clinical insights into the influence of time of day at which high-intensity interval exercise was undertaken, in individuals with type 2 diabetes under a controlled food intake regimen. The primary outcome was the difference in mean 24 h glucose levels between morning and afternoon high-intensity interval exercise bouts. The secondary outcome was the glycaemic response to the meal intervention. Our findings demonstrate that, in women and men with type 2 diabetes, morning high-intensity interval exercise results in greater post-exercise glucose elevations compared with afternoon exercise. These elevations are accompanied by higher levels of NT-proBNP and CRP during morning exercise, suggesting that low-grade inflammation in the presence of high cortisol levels amplifies glycaemic excursions in individuals with type 2 diabetes.

Previous research indicated that morning exercise worsened glucose management in men with type 2 diabetes when training was performed over a 2-week period (three exercise sessions per week), whereas afternoon exercise improved glucose levels [4]. Similarly, a recent study employing moderate-intensity exercise (brisk walking) in individuals with type 2 diabetes found slightly lower glucose levels following afternoon sessions, despite no significant differences in 24 h CGM-measured mean glucose [25]. However, the exercise intensity in the latter study was lower than the high-intensity interval exercise protocols used here, underscoring the potential influence of exercise intensity on glycaemia. Studies that demonstrated a greater beneficial effect on glucose management of exercise in the afternoon compared with the morning for individuals with type 2 diabetes all involved moderate- to high-intensity exercise [4–6]. These findings highlight the importance of both exercise timing and intensity as critical factors influencing glycaemic responses, and suggest that higher-intensity protocols may amplify the impact of circadian rhythms on blood glucose management in individuals with type 2 diabetes.

The observed differences between morning and afternoon exercise may be attributed to circadian fluctuations in hormonal and inflammatory markers. The levels of cortisol and proinflammatory cytokines peak during the morning sleep–wake transition and decline throughout the day [26–29]. Cortisol promotes hepatic glucose production, while inflammatory markers such as CRP can induce hepatic insulin resistance, further driving hyperglycaemia [30, 31]. In the present study, levels of inflammatory markers were higher in the morning among type 2 diabetic cohorts, whereas NT-proBNP and CRP were elevated in the afternoon for participants without type 2 diabetes. This divergence may reflect a heightened inflammatory profile in individuals with type 2 diabetes, particularly during morning hours [32]. Interestingly, women without type 2 diabetes exhibited greater post-exercise glucose elevations following afternoon high-intensity interval exercise compared with baseline, aligning with the observed higher NT-proBNP and CRP levels during afternoon sessions. These findings suggest metabolic health-dependent differences in inflammatory and glycaemic responses to exercise timing.

Adherence to regular eating patterns rapidly improved glucose levels and reduced glycaemic variability in participants with type 2 diabetes. These benefits were evident on the first day of dietary interventions, and were lost upon discontinuation of the structured eating protocol. Both women and men with type 2 diabetes exhibited lower glucose levels during controlled eating and exercise days, particularly at night. Our findings align with prior research emphasising the importance of consistent meal timing for glycaemic control in individuals at risk of type 2 diabetes [18]. Additionally, evidence suggests that a high meal frequency may be more effective than fewer meals in improving glucose regulation in individuals with type 2 diabetes and impaired glucose tolerance [33]. This effect may be attributed to reduced glucose fluctuations when eating smaller, more frequent meals, thereby alleviating metabolic stress and preserving insulin-producing cells. We hypothesised that the exercise bout would improve 24 h glucose management. However, the substantial glycaemic improvement achieved through consistent meal timing may have masked the additional benefits of exercise on 24 h glucose management by limiting the potential for further enhancements. Overall, our results highlight the importance of consistent meal timing to optimise blood glucose regulation in individuals with type 2 diabetes.

Notably, women, irrespective of type 2 diabetes status, exhibited better overall metabolic profiles than men. The HbA_1c_, fasting glucose, insulin and HOMA-IR values were consistently more favourable among women. Additionally, non-diabetic women had better lipid profiles than non-diabetic men, whereas this was not the case for women with type 2 diabetes compared with men with type 2 diabetes, except for triglyceride values. These differences may partly reflect persistent hormonal influences, as oestrogen levels confer vasoprotective effects, reducing inflammation, oxidative stress and cardiovascular risk [12, 34, 35]. However, in women with type 2 diabetes, these protective mechanisms are often disrupted by hyperglycaemia and increased oxidative stress [36, 37]. These findings underscore the complex interplay between sex-specific hormonal influences and metabolic health, highlighting the need for tailored approaches to managing type 2 diabetes that account for differences in biological and cardiovascular risk profiles.

This study has several limitations. The predominantly older study population lacked representation from diverse ancestries and ethnic groups, potentially limiting the generalisability of our findings. Additionally, the absence of body composition analysis precluded a more detailed assessment of fat and lean mass, which could have provided valuable context for interpreting metabolic responses. Our findings are also limited to acute high-intensity interval exercise, and the results may not be reflective of the impact of a long-term training programme. Moreover, the impact of the timing of low- or moderate-intensity exercise on glucose management was not explored. Furthermore, while participants consumed standardised meals and snacks, these were not tailored to individual energy and macronutrient needs, potentially introducing variability in dietary impacts. Related to this, potential differences in the digestive process, including the rate of gastric emptying, may have contributed to the differences in glycaemic responses, as these processes exhibit circadian variations. Nonetheless, the meals provided to the study participants were standardised for energy and macronutrient composition between trials.

In conclusion, this clinical crossover trial highlights the importance of the timing of high-intensity interval exercise for individuals with type 2 diabetes. Afternoon high-intensity interval exercise was more beneficial than morning sessions in mitigating glycaemic spikes, offering stable glucose management during and after exercise. The diurnal hormonal milieu in individuals with type 2 diabetes, characterised by elevated morning levels of cortisol and inflammatory markers, may adversely affect the blood glucose response to exercise. Our findings also underscore the importance of regular eating patterns in optimising glycaemic management, with structured meal timing reducing postprandial glucose excursions and glycaemic variability. These results emphasise the need to incorporate tailored exercise timing and consistent dietary guidance into diabetes management programmes. Such strategies could enhance metabolic control and reduce the risk of diabetes-related complications, forming a cornerstone of comprehensive care for individuals with type 2 diabetes.

## Supplementary Information

Below is the link to the electronic supplementary material.

ESM Figs (PDF 1001 KB)

## Data Availability

All source data are available from the corresponding author on request.

## Abbreviations

CGM: Continuous glucose monitor(ing)
CRP: C-reactive protein
MAGE: Mean amplitude of glycaemic excursions
NT-proBNP: N-terminal prohormone of brain natriuretic peptide

## References

[1] Bull FC, Al-Ansari SS, Biddle S et al (2020) World Health Organization 2020 guidelines on physical activity and sedentary behaviour. Br J Sports Med 54:1451–1462. 10.1136/bjsports-2020-102955 33239350 10.1136/bjsports-2020-102955 PMC7719906

[2] Davies MJ, Aroda VR, Collins BS et al (2022) Management of hyperglycaemia in type 2 diabetes, 2022. A consensus report by the American Diabetes Association (ADA) and the European Association for the Study of Diabetes (EASD). Diabetologia 65:1925–1966. 10.1007/s00125-022-05787-2 36151309 10.1007/s00125-022-05787-2 PMC9510507

[3] Qian J, Scheer F (2016) Circadian system and glucose metabolism: implications for physiology and disease. Trends Endocrinol Metab 27:282–293. 10.1016/j.tem.2016.03.005 27079518 10.1016/j.tem.2016.03.005 PMC4842150

[4] Savikj M, Gabriel BM, Alm PS et al (2019) Afternoon exercise is more efficacious than morning exercise at improving blood glucose levels in individuals with type 2 diabetes: a randomised crossover trial. Diabetologia 62:233–237. 10.1007/s00125-018-4767-z 30426166 10.1007/s00125-018-4767-z PMC6323076

[5] Mancilla R, Brouwers B, Schrauwen-Hinderling VB, Hesselink MKC, Hoeks J, Schrauwen P (2021) Exercise training elicits superior metabolic effects when performed in the afternoon compared to morning in metabolically compromised humans. Physiol Rep 8:e14669. 10.14814/phy2.14669 33356015 10.14814/phy2.14669 PMC7757369

[6] Moholdt T, Parr EB, Devlin BL, Debik J, Giskeodegard G, Hawley JA (2021) The effect of morning vs evening exercise training on glycaemic control and serum metabolites in overweight/obese men: a randomised trial. Diabetologia 64:2061–2076. 10.1007/s00125-021-05477-5 34009435 10.1007/s00125-021-05477-5 PMC8382617

[7] Munan M, Dyck RA, Houlder S et al (2020) Does exercise timing affect 24-hour glucose concentrations in adults with type 2 diabetes? A follow up to the exercise-physical activity and diabetes glucose monitoring study. Can J Diabetes 44:711-718.e711. 10.1016/j.jcjd.2020.05.012 32878737 10.1016/j.jcjd.2020.05.012

[8] Teo SYM, Kanaley JA, Guelfi KJ, Marston KJ, Fairchild TJ (2020) The effect of exercise timing on glycemic control: a randomized clinical trial. Med Sci Sports Exerc 52:323–334. 10.1249/MSS.0000000000002139 31479004 10.1249/MSS.0000000000002139

[9] Pillon NJ, Smith JAB, Alm PS et al (2022) Distinctive exercise-induced inflammatory response and exerkine induction in skeletal muscle of people with type 2 diabetes. Sci Adv 8:eabo3192. 10.1126/sciadv.abo3192 36070371 10.1126/sciadv.abo3192 PMC9451165

[10] Miyamoto T, Fukuda K, Watanabe K, Hidaka M, Moritani T (2015) Gender difference in metabolic responses to surface electrical muscle stimulation in type 2 diabetes. J Electromyogr Kinesiol 25:136–142. 10.1016/j.jelekin.2014.06.013 25066515 10.1016/j.jelekin.2014.06.013

[11] Kanaley JA, Goulopoulou S, Franklin R et al (2012) Exercise training improves hemodynamic recovery to isometric exercise in obese men with type 2 diabetes but not in obese women. Metabolism 61:1739–1746. 10.1016/j.metabol.2012.07.014 22902004 10.1016/j.metabol.2012.07.014 PMC3504623

[12] O’Connor E, Kiely C, O’Shea D, Green S, Egana M (2012) Similar level of impairment in exercise performance and oxygen uptake kinetics in middle-aged men and women with type 2 diabetes. Am J Physiol Regul Integr Comp Physiol 303:R70–R76. 10.1152/ajpregu.00012.2012 22538515 10.1152/ajpregu.00012.2012

[13] American Diabetes Association Professional Practice Committee (2024) Summary of revisions: standards of care in diabetes – 2025. Diabetes Care 48(1 Suppl 1):S6–S13. 10.2337/dc25-SREV 10.2337/dc25-SREV PMC11635056 39651984

[14] Mattson MP, Allison DB, Fontana L et al (2014) Meal frequency and timing in health and disease. Proc Natl Acad Sci USA 111:16647–16653. 10.1073/pnas.1413965111 25404320 10.1073/pnas.1413965111 PMC4250148

[15] Kashiwagi K, Inaishi J, Kinoshita S et al (2023) Assessment of glycemic variability and lifestyle behaviors in healthy nondiabetic individuals according to the categories of body mass index. PLoS One 18:e0291923. 10.1371/journal.pone.0291923 37792730 10.1371/journal.pone.0291923 PMC10550127

[16] Kharmats AY, Popp C, Hu L et al (2023) A randomized clinical trial comparing low-fat with precision nutrition-based diets for weight loss: impact on glycemic variability and HbA1c. Am J Clin Nutr 118:443–451. 10.1016/j.ajcnut.2023.05.026 37236549 10.1016/j.ajcnut.2023.05.026 PMC10447469

[17] Akasaka T, Sueta D, Tabata N et al (2017) Effects of the mean amplitude of glycemic excursions and vascular endothelial dysfunction on cardiovascular events in nondiabetic patients with coronary artery disease. J Am Heart Assoc 6(5):e004841. 10.1161/JAHA.116.004841 28446494 10.1161/JAHA.116.004841 PMC5524064

[18] Zhao L, Teong XT, Liu K et al (2022) Eating architecture in adults at increased risk of type 2 diabetes: associations with body fat and glycaemic control. Br J Nutr 128:324–333. 10.1017/S0007114521002944 34348822 10.1017/S0007114521002944

[19] Socialstyrelsen (2011) Quality and efficiency of diabetes care in Sweden – aspects of equity in a national quality register. Available from https://www.socialstyrelsen.se/globalassets/sharepoint-dokument/artikelkatalog/statistik/2014-3-18.pdf

[20] Gaskill SE, Ruby BC, Walker AJ, Sanchez OA, Serfass RC, Leon AS (2001) Validity and reliability of combining three methods to determine ventilatory threshold. Med Sci Sports Exerc 33:1841–1848. 10.1097/00005768-200111000-00007 11689733 10.1097/00005768-200111000-00007

[21] Borg GAV (1982) Psychophysical bases of perceived exertion. Med Sci 14:377–381. 10.1249/00005768-198205000-00012 7154893

[22] Lang RM, Badano LP, Mor-Avi V et al (2015) Recommendations for cardiac chamber quantification by echocardiography in adults: an update from the American Society of Echocardiography and the European Association of Cardiovascular Imaging. J Am Soc Echocardiogr 28:1-39.e14. 10.1093/ehjci/jev014 25559473 10.1016/j.echo.2014.10.003

[23] Broll S, Urbanek J, Buchanan D et al (2021) Interpreting blood glucose data with R package iglu. PLoS One 16:e0248560. 10.1371/journal.pone.0248560 33793578 10.1371/journal.pone.0248560 PMC8016265

[24] Harris PA, Taylor R, Thielke R, Payne J, Gonzalez N, Conde JG (2009) Research electronic data capture (REDCap) – -a metadata-driven methodology and workflow process for providing translational research informatics support. J Biomed Inform 42:377–381. 10.1016/j.jbi.2008.08.010 18929686 10.1016/j.jbi.2008.08.010 PMC2700030

[25] Niu WC, Liu C, Liu K et al (2024) The effect of different times of day for exercise on blood glucose fluctuations. Prim Care Diabetes 18:427–434. 10.1016/j.pcd.2024.06.004 38897914 10.1016/j.pcd.2024.06.004

[26] Liu PY (2024) Rhythms in cortisol mediate sleep and circadian impacts on health. Sleep 47(9):zsae151. 10.1093/sleep/zsae151 38963818 10.1093/sleep/zsae151 PMC11381560

[27] Koch CE, Leinweber B, Drengberg BC, Blaum C, Oster H (2017) Interaction between circadian rhythms and stress. Neurobiol Stress 6:57–67. 10.1016/j.ynstr.2016.09.001 28229109 10.1016/j.ynstr.2016.09.001 PMC5314421

[28] Walker WH 2nd, Walton JC, DeVries AC, Nelson RJ (2020) Circadian rhythm disruption and mental health. Transl Psychiatry 10:28. 10.1038/s41398-020-0694-0 32066704 10.1038/s41398-020-0694-0 PMC7026420

[29] McAlpine CS, Swirski FK (2016) Circadian influence on metabolism and inflammation in atherosclerosis. Circ Res 119:131–141. 10.1161/CIRCRESAHA.116.308034 27340272 10.1161/CIRCRESAHA.116.308034 PMC4922503

[30] Sharabi K, Tavares CD, Rines AK, Puigserver P (2015) Molecular pathophysiology of hepatic glucose production. Mol Aspects Med 46:21–33. 10.1016/j.mam.2015.09.003 26549348 10.1016/j.mam.2015.09.003 PMC4674831

[31] Groeger M, Matsuo K, HeidaryArash E et al (2023) Modeling and therapeutic targeting of inflammation-induced hepatic insulin resistance using human iPSC-derived hepatocytes and macrophages. Nat Commun 14:3902. 10.1038/s41467-023-39311-w 37400454 10.1038/s41467-023-39311-w PMC10318012

[32] Yu R, Tian L, Ding Y, Gao Y, Li D, Tang Y (2019) Correlation between inflammatory markers and impaired circadian clock gene expression in type 2 diabetes mellitus. Diabetes Res Clin Pract 156:107831. 10.1016/j.diabres.2019.107831 31476346 10.1016/j.diabres.2019.107831

[33] Papakonstantinou E, Kontogianni MD, Mitrou P et al (2018) Effects of 6 *vs* 3 eucaloric meal patterns on glycaemic control and satiety in people with impaired glucose tolerance or overt type 2 diabetes: a randomized trial. Diabetes Metab 44:226–234. 10.1016/j.diabet.2018.03.008 29680359 10.1016/j.diabet.2018.03.008

[34] Mikkola TS, Clarkson TB (2002) Estrogen replacement therapy, atherosclerosis, and vascular function. Cardiovasc Res 53:605–619. 10.1016/S0008-6363(01)00466-7 11861031 10.1016/s0008-6363(01)00466-7

[35] Xing D, Nozell S, Chen YF, Hage F, Oparil S (2009) Estrogen and mechanisms of vascular protection. Arterioscler Thromb Vasc Biol 29:289–295. 10.1161/ATVBAHA.108.182279 19221203 10.1161/ATVBAHA.108.182279 PMC2700771

[36] Simon AB, Derella CC, Blackburn M et al (2023) Endogenous estradiol contributes to vascular endothelial dysfunction in premenopausal women with type 1 diabetes. Cardiovasc Diabetol 22:243. 10.1186/s12933-023-01966-6 37679748 10.1186/s12933-023-01966-6 PMC10486136

[37] Sridharan S (2010) Are there differences in cardiovascular and metabolic risk profiles among men and women with type 2 diabetes? A cross-sectional analysis. Diabet Med 27:1212–1214. 10.1111/j.1464-5491.2010.03077.x 20854390 10.1111/j.1464-5491.2010.03077.x

