# Supplementary material for: Inflammatory markers and blood glucose are higher after morning vs afternoon exercise in type 2 diabetes
Source: Diabetologia. 2025 Jun 28;68(9):2023–35. doi: 10.1007/s00125-025-06477-5 (PMC12361293; doi:10.1007/s00125-025-06477-5)
Supplement: Supplementary file 1 — ESM Figs (PDF 1001 KB) [file 125_2025_6477_MOESM1_ESM.pdf]

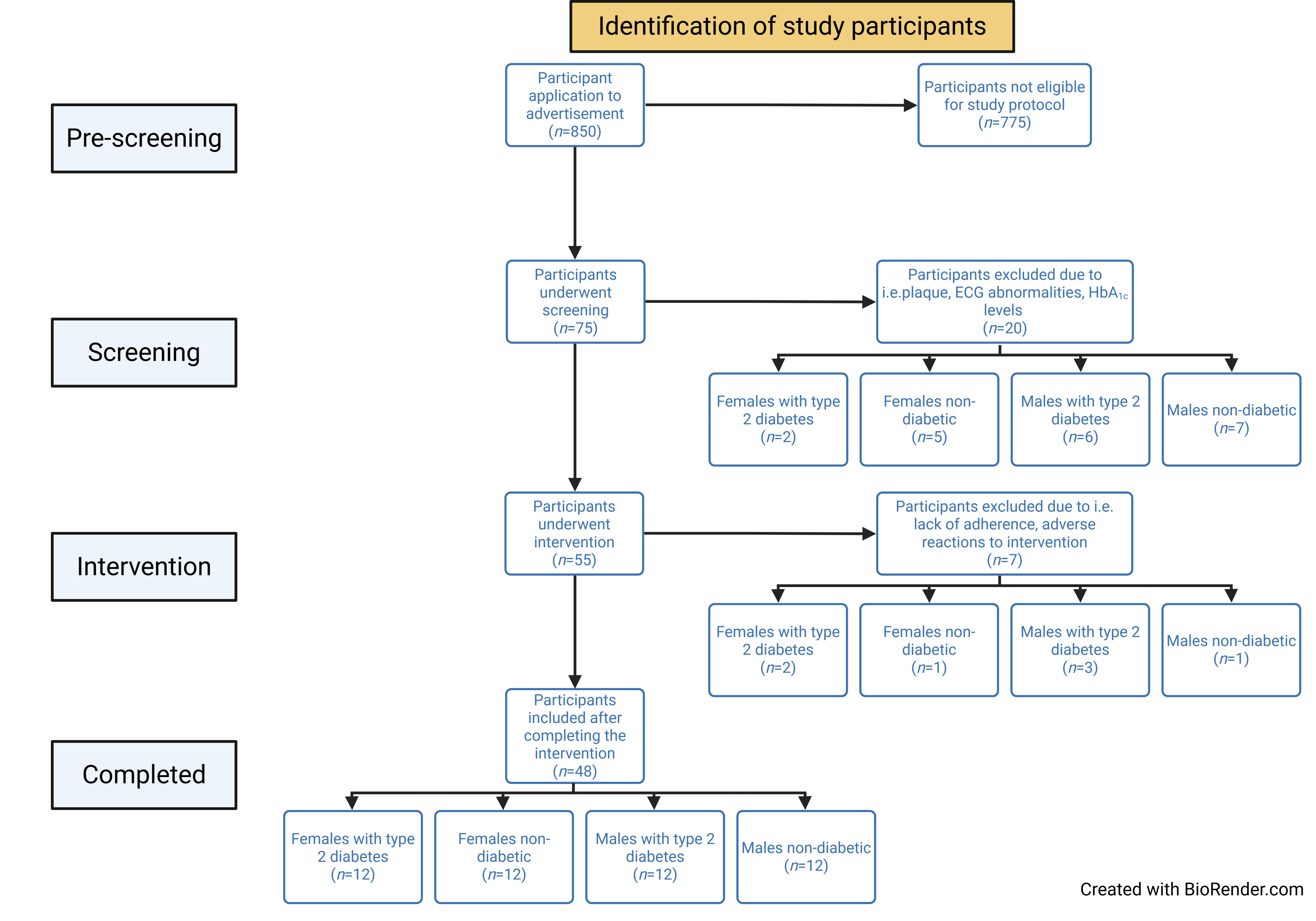
Identification of study participants
Pre-screening
Participant application to advertisement (n=850)
Participants not eligible for study protocol (n=775)
Screening
Participants underwent screening (n=75)
Participants excluded due to i.e.plaque, ECG abnormalities, HbA1c levels (n=20)
Females with type 2 diabetes (n=2)
Females non-diabetic (n=5)
Males with type 2 diabetes (n=6)
Males non-diabetic (n=7)
Intervention
Participants underwent intervention (n=55)
Participants excluded due to i.e. lack of adherence, adverse reactions to intervention (n=7)
Females with type 2 diabetes (n=2)
Females non-diabetic (n=1)
Males with type 2 diabetes (n=3)
Males non-diabetic (n=1)
Completed
Participants included after completing the intervention (n=48)
Females with type 2 diabetes (n=12)
Females non-diabetic (n=12)
Males with type 2 diabetes (n=12)
Males non-diabetic (n=12)
Created with BioRender.com

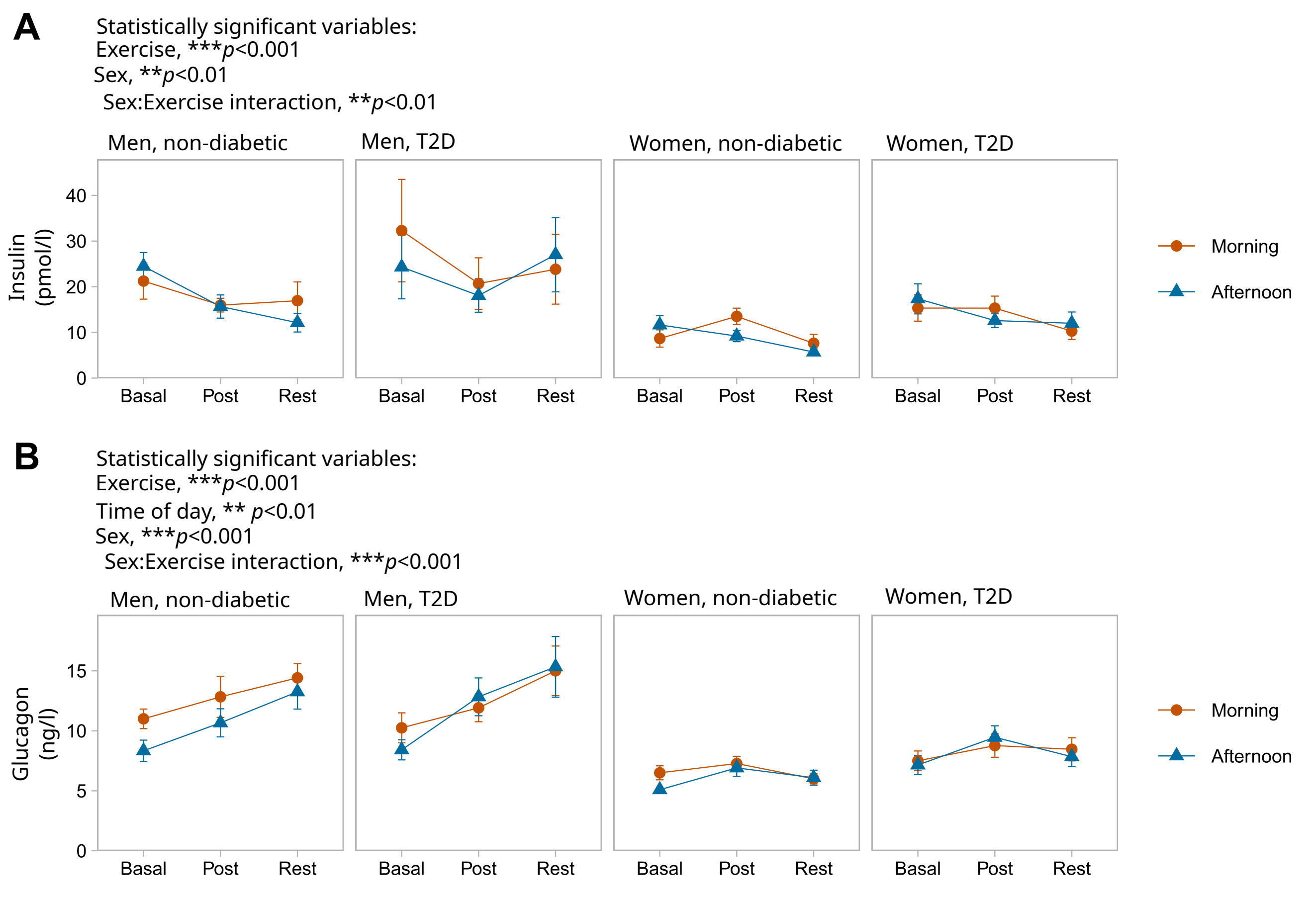
A
Statistically significant variables:
Exercise, ***p<0.001
Sex, **p<0.01
Sex:Exercise interaction, **p<0.01
Men, non-diabetic
Men, T2D
Women, non-diabetic
Women, T2D
Insulin (pmol/l)
0
10
20
30
40
Basal
Post
Rest
Morning
Afternoon
B
Statistically significant variables:
Exercise, ***p<0.001
Time of day, ** p<0.01
Sex, ***p<0.001
Sex:Exercise interaction, ***p<0.001
Men, non-diabetic
Men, T2D
Women, non-diabetic
Women, T2D
Glucagon (ng/l)
0
5
10
15
Basal
Post
Rest
Morning
Afternoon

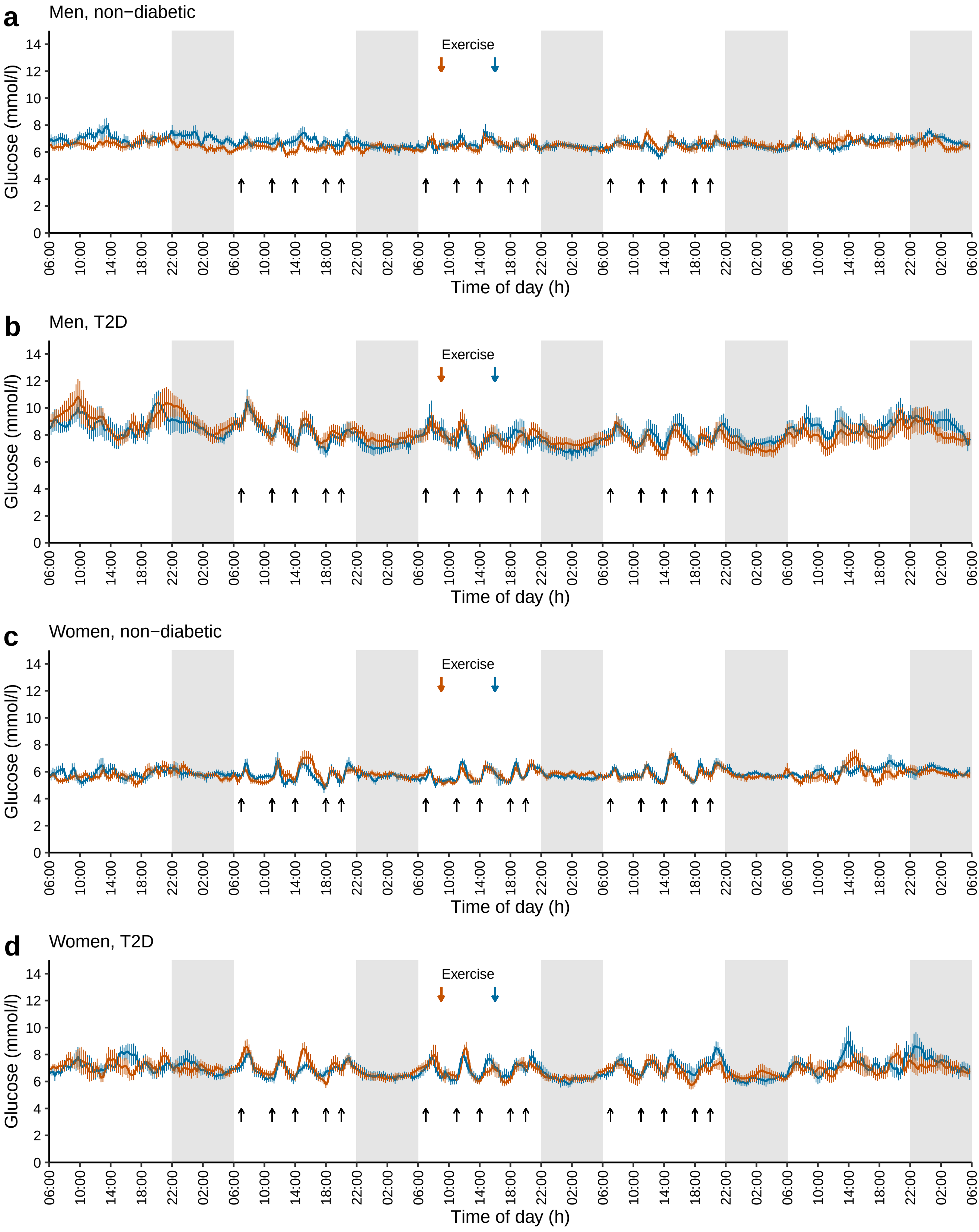
a
Men, non−diabetic
Exercise
Glucose (mmol/l)
0
2
4
6
8
10
12
14
06:00
10:00
14:00
18:00
22:00
02:00
06:00
10:00
14:00
18:00
22:00
02:00
06:00
10:00
14:00
18:00
22:00
02:00
06:00
10:00
14:00
18:00
22:00
02:00
06:00
10:00
14:00
18:00
22:00
02:00
06:00
Time of day (h)
b
Men, T2D
Exercise
Glucose (mmol/l)
Time of day (h)
c
Women, non−diabetic
Exercise
Glucose (mmol/l)
Time of day (h)
d
Women, T2D
Exercise
Glucose (mmol/l)
Time of day (h)
